# Photocatalytic Degradation of Methylene Blue Dye using PANI‐CuFe_2_O_4_ Nano Composite

**DOI:** 10.1002/gch2.202400179

**Published:** 2024-11-15

**Authors:** Tisa Rani Saha, Md. Ahsan Habib, S. M. Imran Ali, Jannatul Naime, Md. Mahiuddin, Shaheen M. Sarkar, Md. Abu Rayhan Khan, Kaykobad Md Rezaul Karim

**Affiliations:** ^1^ Chemistry Discipline Khulna University Khulna 9208 Bangladesh; ^2^ Department of Applied Science Technological University of the Shannon Midlands Midwest, Moylish Limerick V94 EC5T Ireland

**Keywords:** methylene blue, nanocomposite, photocatalyst

## Abstract

The present perspective accentuates the synthesis of PANI‐CuFe_2_O_4_ (PCF) nanocomposite, and photocatalytic degradation of methylene blue (MB) dye using a synthesized composite. The stable PCF is confirmed and characterized by analytical techniques, namely, fourier transform infrared (FTIR) and X‐ray photoelectron (XPS) spectroscopy, X‐ray diffraction (XRD), energy‐dispersive X‐ray (EDX), scanning electron microscopy (SEM), and vibrating sample magnetometry (VSM) analysis. The synthesized PCF nanocomposites are significantly crystalline in nature, having magnetic saturation of 10.47 emu g^−1^, and monoclinic crystalline structure as well as the size of nanocomposite is 39.54 nm verified by XRD pattern. SEM analysis revealed a regular porous and rough surface of nanocomposite. In addition, the nanocomposite divulged the remarkable efficient elimination of MB dye with maximum removal of 96% with good fitting of Langmuir isotherm, indication of monolayer formation on the catalyst surface through the interaction between nanocomposite and dye molecule. The adsorption kinetics bolstered the pseudo‐second‐order kinetic model, suggesting the adsorption process proceeded by chemisorption. The most notable feature of the nanocomposite is the reusability and good stability after several cycles, maintaining 90% after five cycles.

## Graphical abstract

This present research focus on the synthesis of PANI‐CuFe_2_O_4_ (PCF) nanocomposite for the photocatalytic degradation of toxic methylene blue (MB) dye. Nanocomposite showed significant elimination of MB dye with maximum removal of 96% with the best fitting of Langmuir isotherm, an indication of monolayer formation on catalyst surface through the interaction between nanocomposite and dye molecule.

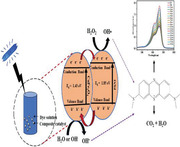

## 1 Introduction

Water, an integral element of the environment, is frequently defiled owing to the excessive application of fertilizers, pesticides, chemicals, and dyes in farmland as well as industries, resulting in getting mixed with water bodies.^[^ ^1^ ^]^ Of them, organic dyes extensively used in textile, leather, cosmetics, printing, paper, and food processing industries exert detrimental effects on biota since the organic dyes cause a variety of illnesses including the poisoning of the central nervous system, skin rashes, kidney and liver damage, and blood abnormalities.^[^ ^2^, ^3^ ^]^ Consequently, the foremost and supreme concern is to protect the aqueous medium from the adverse effects of dyes. The purport, however, is implemented through a myriad number of analytical techniques, including oxidation, oxidation process, electrochemical destruction, Fenton reaction, ozonation, irradiation, filtration, ion exchange, reverse osmosis, photochemical, and ultraviolet irradiation.^[^ ^4^ ^]^ In this regard, the relation between surface area and amount of adsorbent highly influences the removal of organic dyes from aqueous medium [Reference]. Nanotechnology is the suitable solution to eliminate organic dye because of its remarkable cost reduction, easy way to synthesize, and capability to amass large volumes of pollutants.

Over the last few decades, semiconducting magnetic ferrites of different metals such as Co, Cu, Ni, Mn, Zn, Sr etc. have been employed in the form of nano size for the distillation of waste water owing to distinctive properties, for instance, spinel structure, composition, high stability, recyclability, and low bandgap energy.^[^ ^5^ ^]^ This research highlights the synthesis of nanocomposite (PANI‐CuFe_2_O_4_), a promising photocatalyst, for the photodegradation of MB as a model dye. The major component of the composite is copper ferrite (CuFe_2_O_4_), a semiconductor material, which is less toxic with excellent magnetic properties, photochemical stability, and higher catalytic activity without secondary contamination.^[^ ^6^, ^7^ ^]^ But, the main limitation of CuFe_2_O_4_ is the low catalytic activity owing to the higher recombination rate of electron‐hole (e‐/h+) pairs.^[^ ^8^ ^]^ The annexation of various polymeric materials with CuFe_2_O_4_ upgrades the catalytic performance. Recently, carbon based supporting materials, including graphene, graphitic carbon nitride, activated carbon, and nitrogen‐doped carbon nanotubes have been reported as promising catalysts due to their high surface areas and electron transfer capability.^[^ ^9^ ^]^ Moreover, conducting polymer based nanocomposites are employed with various metal ferrites for the enhancement of catalytic performance of both of them.^[^ ^10^, ^11^ ^]^ Numerous studies have been conducted on the combination of two distinct materials to adjust the bandgap of the photocatalyst to improve the electron‐hole charge separation, which leads to an increase in the responsiveness of the photocatalyst under visible light or solar light irradiation.^[^ ^12^ ^]^ Of them, Polyaniline (PANI), a conducting polymer, has received extensive attention because of its distinctive properties, including p‐type semiconductor with narrow bandgap, electron donor, and hole acceptor leading to effective charge separation, environmentally benign, non‐hazardous, and easy synthesis route. But, poor mechanical characteristics, and low stability have limited the applications of PANI in many environmental applications.^[^ ^8^, ^13^, ^14^ ^]^ However, the incorporation of PANI in CuFe_2_O_4_ ameliorated the catalytic performance in the current study. Moreover, extensive researches have been conducted on composite materials such as CuFe_2_O_4_‐graphene oxide (GO) Fourier transform infrared,^[^ ^15^ ^]^ CuFe_2_O_4_‐TiO_2_‐GO,^[^ ^16^ ^]^ PANI‐NiFe_2_O_4_,^[^ ^11^ ^]^ PANI‐g‐C_3_N_4_,^[^ ^17^ ^]^ Chitosan Grafted‐Polyaniline/Co_3_O_4_,^[^ ^14^ ^]^ Ternary‐CoFe_2_O_4_‐PANI nanocomposite^[^ ^12^ ^]^ that have been used in degrading dyes under different conditions.

The aim of this research was to develop an environmentally friendly, stable, magnetically recoverable, and rapid photocatalytic efficient polyaniline‐copper ferrite photocatalyst for the treatment of industrial dye containing wastewater under visible light irradiation.

## 2 Results and Discussion

### 2.1 Characterization of Nanocomposite

**Figure 1:**
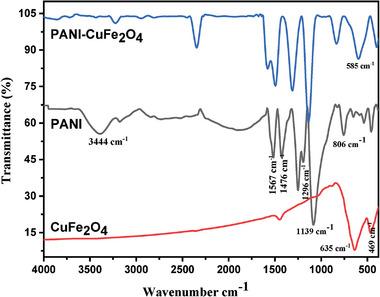
FTIR spectra of CuFe_2_O_4_, PANI, and PANI‐CuFe_2_O_4_ (Conditions: 80 °C and 30 min).

Fourier transform infrared (FTIR) spectra of PANI, CuFe_2_O_4_, and PCF composite has been illustrated in **Figure** **1**. The prominent peaks at 469 and 635 cm^−1^ were attributed to Cu─O and Fe─O intrinsic stretching vibration in octahedral and tetrahedral sites of pure CuFe_2_O_4_ in the absence of any organic moiety, ascertaining the structure of CuFe_2_O_4_.^[^ ^8^ ^]^ FTIR spectrum of PANI revealed a peak at 806 cm^−1^ ratified out of plane deformation of C─H bond in benzene ring, and the peak at 1139 cm^−1^ was assigned to electronic like absorption of N═Q═N (where Q denotes the quinoid ring). In addition, the signal at 1296 cm^−1^ was ascertained the presence of stretching vibration of C─N in the benzenoid ring, whilst two consecutive peaks having equal intensity at 1476 and 1567cm^−1^ corresponded to the C─C stretching of the benzenoid and quinoid rings, respectively. The additional peak at 3215 cm^−1^ represented the protonated form amine segment in PANI (C─N stretching of the benzenoid ring), and a broad band at 3444 cm^−1^ was assigned to the free N─H stretching vibration mode.^[^ ^18^, ^19^, ^20^ ^]^ Similar characteristic peaks of PANI and CuFe_2_O_4_ at higher frequencies remarked the interaction with CuFe_2_O_4_ moiety. Additionally, blue shift of signal in the range of 806–835, 1296–1306, and 1476–1493 cm^−1^ for bending vibration of C─H in benzenoid, stretching vibration of C─N in the benzenoid ring certainly confirmed nanocomposite formation.^[^ ^21^, ^22^ ^]^

**Figure 2:**
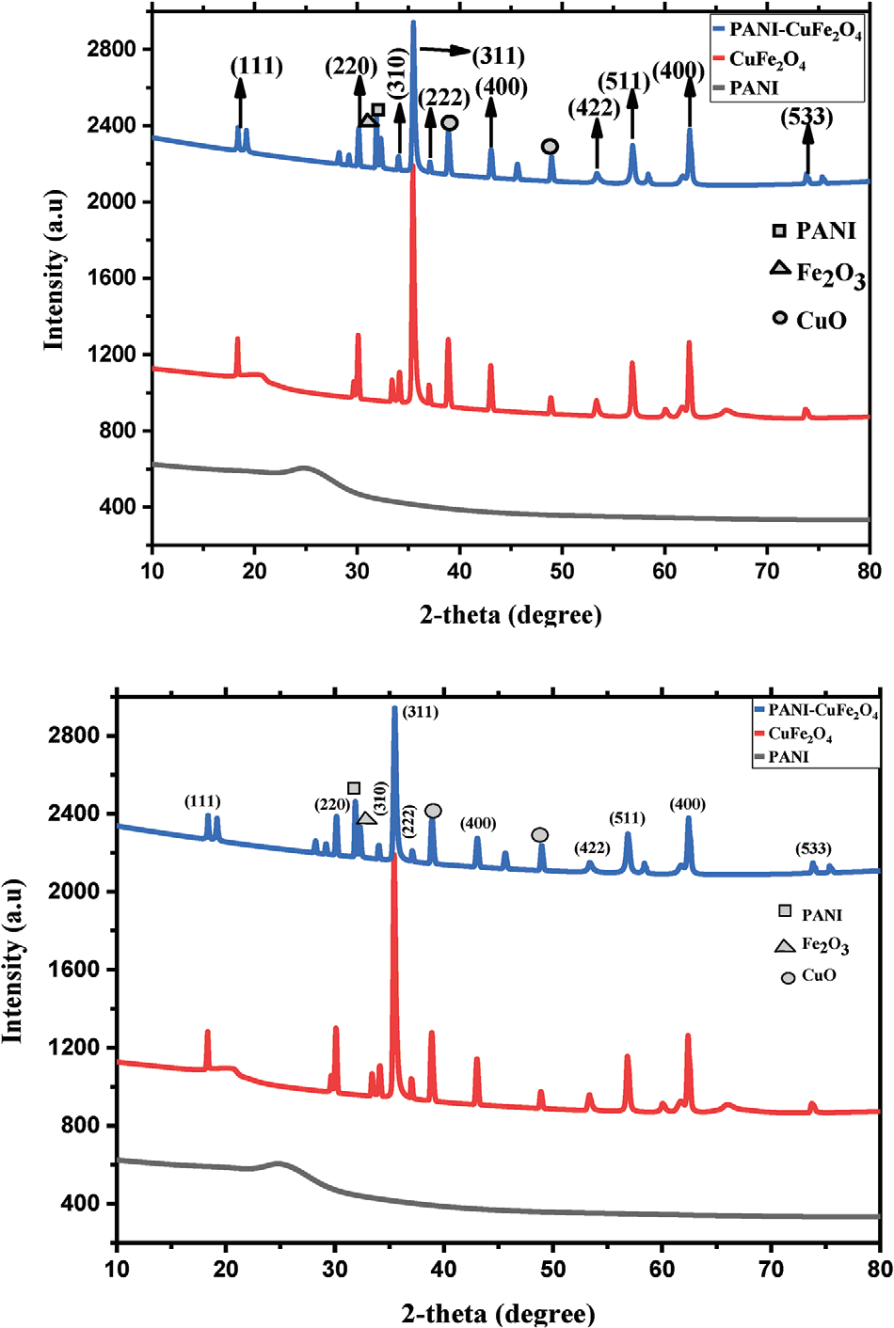
XRD patterns of PANI, CuFe_2_O_4_, and PANI‐CuFe_2_O_4_ (Conditions: 80 °C and 30 min).

Powder X‐ray diffraction (XRD) study expresses the phase and crystalline nature of the nanocomposite. The powder XRD pattern of PANI, CuFe_2_O_4_, and PCF nanocomposite has been illustrated in **Figure** **2**. The pattern expressed ten characteristic responses at 18.35°, 30.08°, 33.39°, 35.42°, 36.99°, 43.00°, 53.33°, 56.81°, 62.38°, and 73.72°, corresponding to Miller index of CuFe_2_O_4_crystal of (111), (220), (310), (311), (222), (320), (400), (420), (422), (511), (400), and (533) respectively, remarking cubic structure of CuFe_2_O_4_ (JCPDS file no: 00‐025‐0283).^[^ ^23^ ^]^ Additionally, monoclinic structure of CuO was identified in the presence of (111) and (202) lattice planes, attributing to 38.82° and 48.86°, respectively.^[^ ^24^ ^]^ The synthesis of nanocomposite was ascertained through the peak at (2θ) 18.37°, 30.13°, 32.29°, 33.98°, 35.49°, 37.08°, 38.86°, 43.04°, 48.96, 53.35°, 56.85°, 62.43°, and 73.83°, supported by JCPDS file no: 1‐074‐8585. In addition, extra peaks at 31° and 32.29° described the presence of α‐Fe_2_O_3_ and PANI, respectively.^[^ ^21^, ^22^, ^25^ ^]^ The XRD pattern of PANI reveals a peak ≈25°, which can be attributed to the scattering from PANI chains at interplanar spacing and indicates that PANI homopolymer possesses a certain degree of crystallinity.^[^ ^26^ ^]^ The presence of peak at 32.29° is considered as the characteristic peak of PANI in the PCF nanocomposite, because it was not present in pure CuFe_2_O_4_ nanoparticles. In addition, it was noted that the diffraction peak of PCF shifts to a higher 2θ value when compared to both pristine PANI and CuFe_2_O_4_, attributed to the interactions occurring between them during the formation of the composite, which also well aligns with the references.^[^ ^21^, ^22^ ^]^ The presence of CuO and Fe_2_O_3_ as secondary phases is likely a result of the synthesis conditions, such as the pH and calcination temperature, which can influence the partial oxidation of copper and iron during the synthesis of CuFe_2_O_4_, leading to the formation of these oxides in CuFe_2_O_4_ and also in the final composite. Additionally, this interaction between the conductive polymer PANI and CuFe_2_O_4_, combined with extended stirring and drying steps in the PCF nanocomposite formation, likely contributes to the formation of CuO and Fe_2_O_3_ in the composite.^[^ ^27^, ^28^ ^]^ The average crystallite size of PANI, CuFe_2_O_4_ and PCF nanocomposite was calculated using Debye–Scherrer formula.^[^ ^29^ ^]^

(1) $$D=\frac{0.9\;\lambda }{\beta \;\cos\theta }$$

Where, D describes average crystal size, λ represents X‐ray wavelength, β represents broadening of the diffraction peak at half maximum, and θ indicates diffraction angle. The average crystallite size of CuFe_2_O_4_ and PCF nanocomposite was calculated as 41.94  and 39.94 nm, respectively.

**Figure 3:**
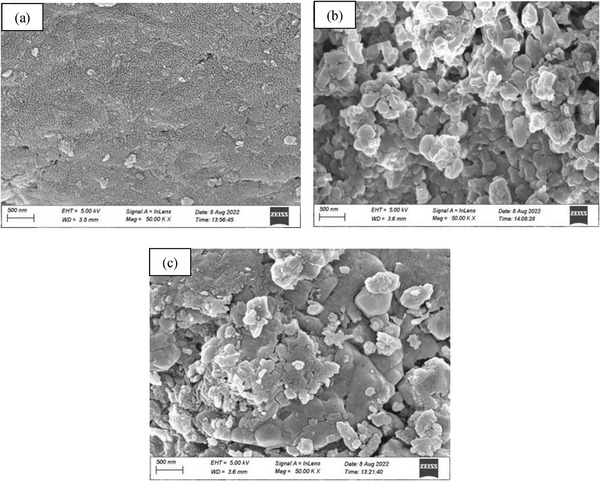
FE‐SEM images of (a) PANI, (B) CuFe_2_O_4_, and (c) PANI‐ CuFe_2_O_4_ (Conditions: 80 °C and 30 min).

Field emission scanning electron microscopy (FE‐SEM) was conducted to carefully investigate the surface morphology and size of the nanocomposite. FE‐SEM image (**Figure** **3**) depicts the surface structure of PANI, CuFe_2_O_4_, and PCF. The image (Figure 3a) shows that the particles are almost sponge in size for PANI, but the surface image (Figure 3b) of pure CuFe_2_O_4_ exhibited the particles are in highly agglomerated form on the surface. However, we predicted the particle size in the range of 46  to 53 nm with well dispersed of CuFe_2_O_4_ on the surface of PANI (Figure 3c).

**Figure 4:**
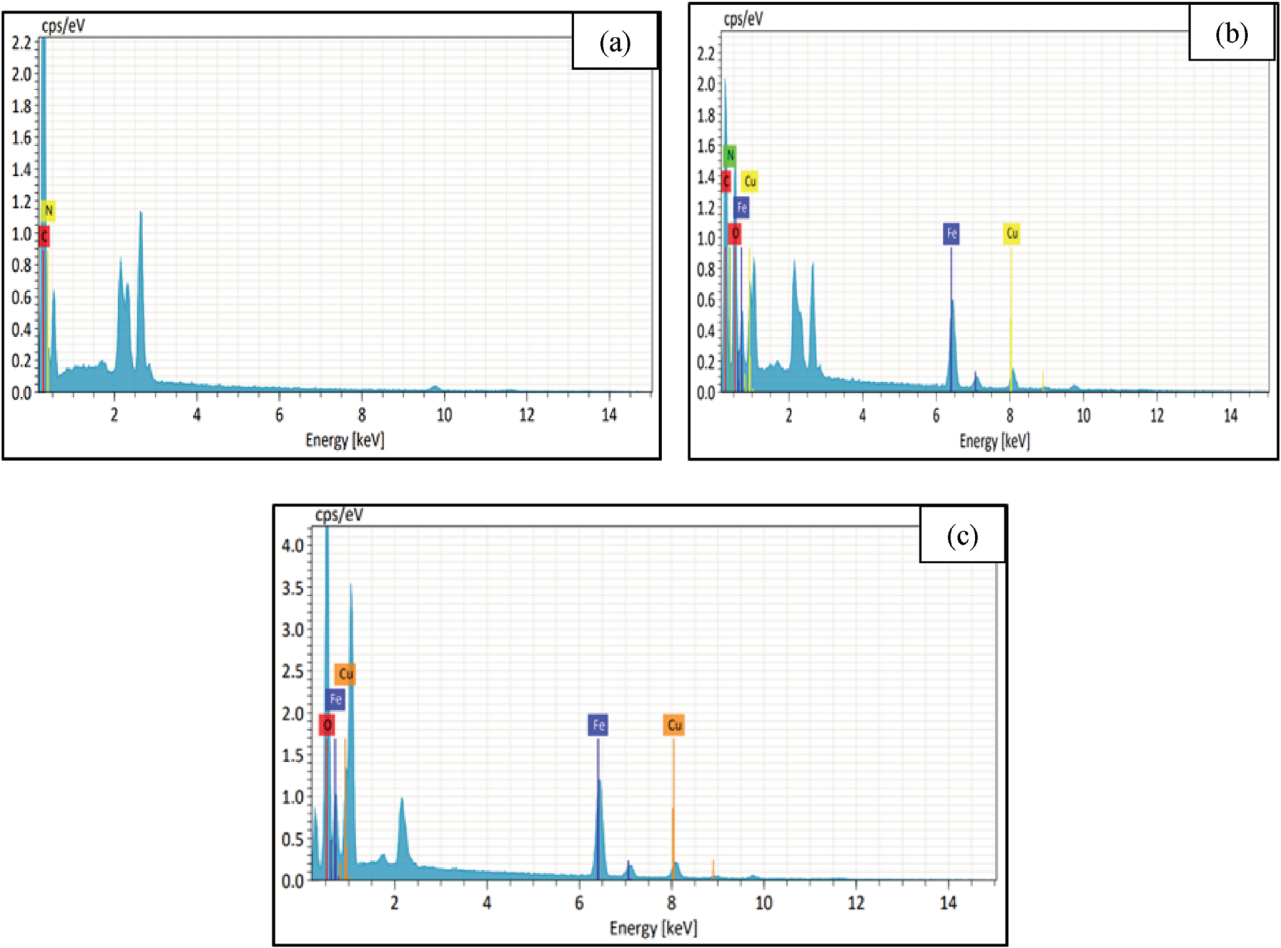
Energy‐dispersive X‐ray spectra of (a) PANI (b) CuFe_2_O_4_, and (c) PANI‐CuFe_2_O_4_ (Conditions: 80 °C and 30 min).

**Figure 5:**
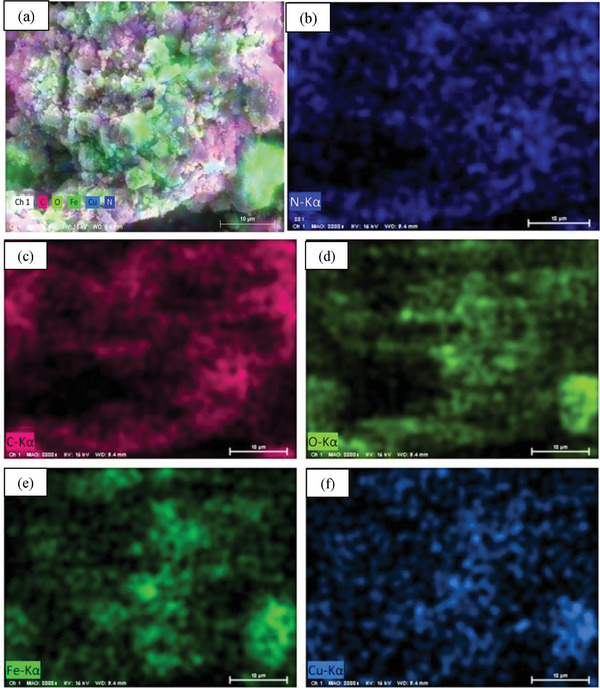
Energy‐dispersive X‐ray photoelectron color mapping of (a) PANI‐CuFe_2_O_4_ (b) N (c) C (d) O (e) Fe, and (f) Cu.

Elemental composition of nanocomposite was estimated using energy‐dispersive X‐ray (EDX) spectroscopy. The EDX spectrum has been shown in **Figure** **4**. The presence of C (59.83%) and N (40.17%) is the indication of proper synthesis of PANI (Figure 4a), and Cu (15.72%), Fe (41.15%), and O (43.12%) confirmed the synthesis of CuFe_2_O_4_ NPs (Figure 4b). The combined signal of C (35.96%), N (11.09%), Cu (27.24%), Fe (16.83%), and O (8.88%) present in Figure 4b ratify the successful formation of nanocomposite. Apart from this, EDX color mapping (**Figure** **5**) also assigns C, N, Cu, Fe, and O element that supports elemental compositions as well as the formation of the nanocomposite.

**Figure 6:**
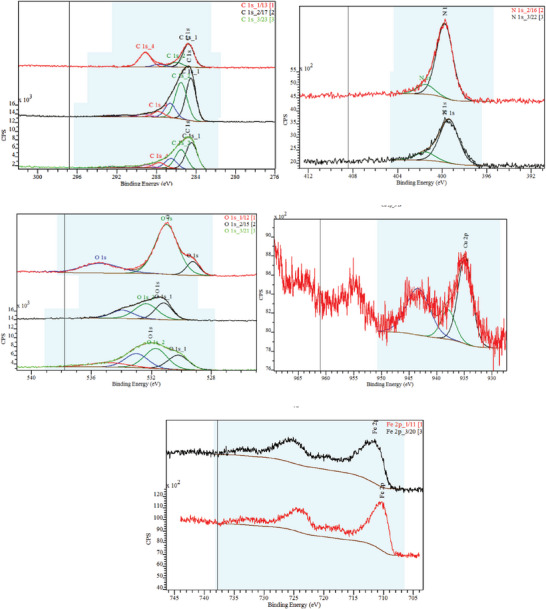
XPS curves of the composite materials.

Oxidation state of the elements present in the catalysts was investigated by the X‐ray photoelectron spectroscopy (XPS) analysis, and the survey spectra for carbon, nitrogen, oxygen, and copper in CuFeO_4_, PANI, and PCF are shown in **Figure** **6**. In the PCF nanocomposite, the deconvolution of the spectrum of C1s revealed five peaks at 284.8, 285.5, 286.5, 287.7, and 289.0 eV for C─C, C─N, C─O, C─O, and O─C═O respectively.^[^ ^30^ ^]^ The N1s spectra resolved into two peaks 399.8 and 401.5 eV due to the quinone diimine nitrogen and protonated nitrogen as the acid doping‐induced N^+^ and proton interaction respectively.^[^ ^31^ ^]^ The Cu2p spectra can be deconvoluted into one peak at 934.8 eV, which might be the result of Cu(0) and Cu(II), respectively, indicating the presence of copper oxide on the Cu surface and the other two peaks are the satellite peaks at 938.0 and 943.4 eV.^[^ ^32^ ^]^ The O1s spectrum shows five peaks at 530.2, 531.7, 532.1, 533.0, and 534.9 eV M─O bond/lattice oxygen, surface adsorbed oxygen/oxygen defect sites, C═O, O^*^NO_2_, and ONO^*^_2_.^[^ ^33^ ^]^ Fe2p spectra represent one major peak at 711.5 eV attributed to 2p_3/2_ for Fe(II) ion.^[^ ^7^ ^]^

**Figure 7:**
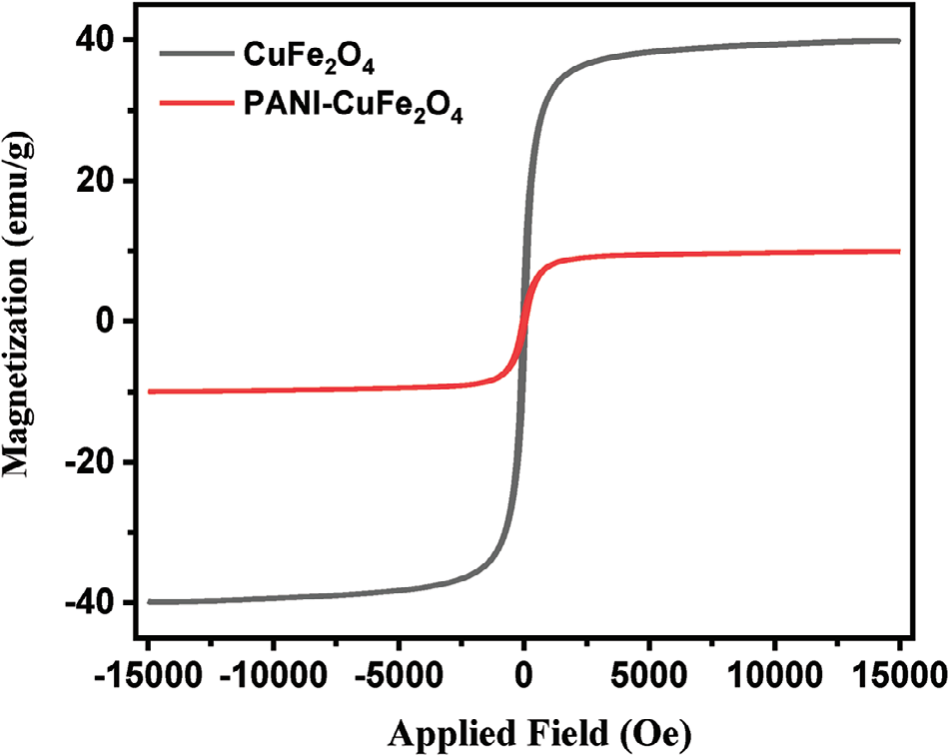
Comparison of magnetization curve of CuFe_2_O_4_ and PANI‐CuFe_2_O_4_ (Conditions: 80 °C and 30 min).

The magnetic behavior of composite material using vibrating sample magnetometry (VSM) and **Figure** **7** show the results of CuFe_2_O_4_ and PCF. The saturation magnetization (M_s_) of the nanocomposite was found to be 41.85 emu g^−1^, while the low values of M_r_ (5.79) and H_c_ (0.79), indicate the super magnetic nature of the composite material. The superparamagnetic materials do not keep their magnetization before or after the removal of the magnetic field, resulting in the prevention of particle aggregation that is significant in photocatalysis owing to available active sites.^[^ ^34^ ^]^ The results expressed that the saturation mass magnetization of the nanocomposite is lower in comparison to naked CuFe_2_O_4_ owing to the annexation of non‐magnetic material (PANI) with CuFe_2_O_4_, resulting in the change of magnetization because of the quenching of the surface moment.^[^ ^22^ ^]^ The saturation mass magnetization (M_s_), remanent mass magnetization (M_r_), and coercivity (H_c_) of CuFe_2_O_4_ and PCF have been enlisted in **Table** **1**.

**Table 1:** Magnetic parameters of CuFe_2_O_4_ and PCF nanocomposite.

Sample	M_s_	M_r_	H_c_
CuFe_2_O_4_	41.85	5.79	0.79
PCF	10.47	1.27	0.29

### 2.2 Photocatalytic Degradation of MB Dye

**Figure 8:**
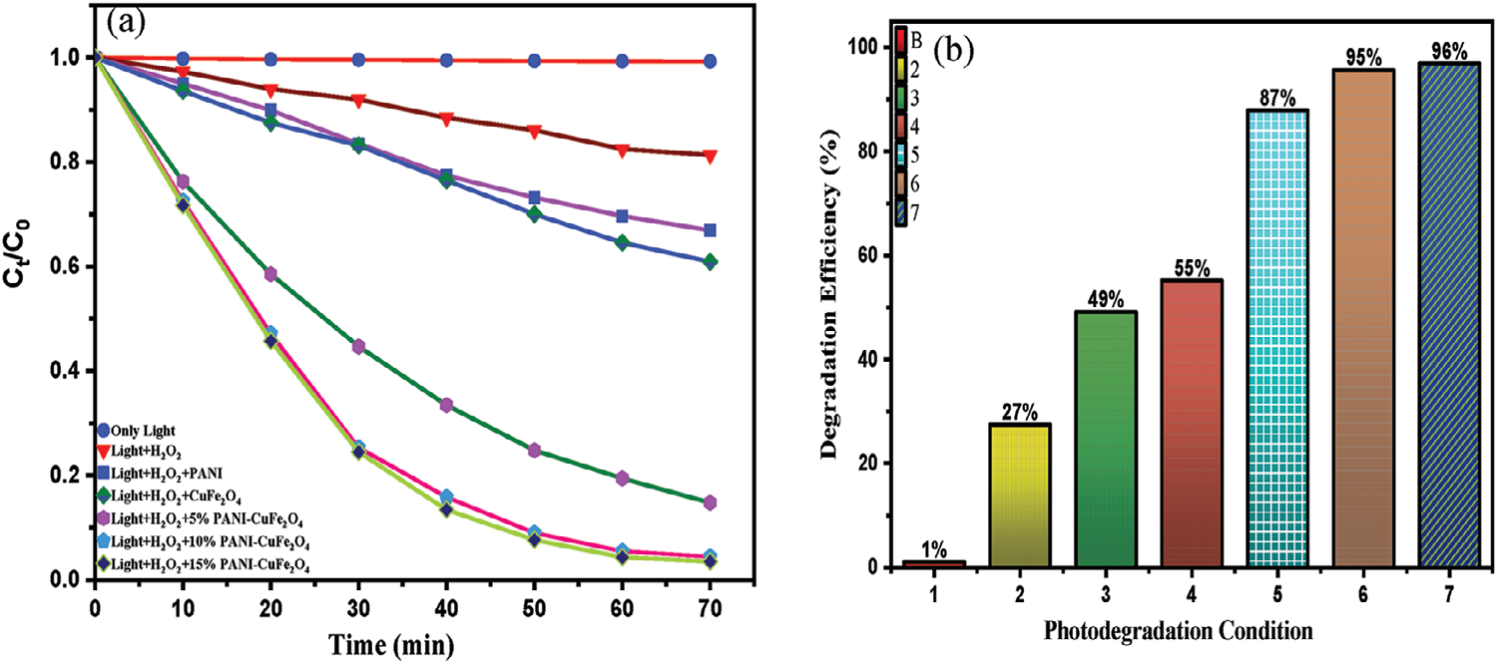
(a) Photodegradation rates of MB dye and (b) degradation efficiency under different conditions.

The maximum efficiency of the photocatalyst was investigated considering several conditions, including only light, (light + H_2_O_2_), and light + H_2_O_2_ + (5%, 10%, and 15%) PCF photo catalyst illustrated in **Figure** **8**. It was notable that a minimal percentage (1%) of dye was degraded in the presence of only light and was uplifted to 27% in the presence of H_2_O_2_. The photocatalyst impacted the reaction, and with the increasing percentage of catalyst (5% to 15%) along with Light + H_2_O_2,_ the efficiency was sharply accelerated to 87% to 96%, owing to the synergistic effect arising from the coupling of PANI and CuFe_2_O_4_ in the nanocomposite.^[^ ^8^ ^]^ On the contrary, the effect of PANI and CuFe_2_O_4_ along with Light + H_2_O_2_ expressed just 49% and 55%, respectively (**Table** **2**).

**Table 2:** Removal of MB dye at various conditions.

MB dye removal condition	Equilibrium time [min]	Removal [%]
Only light	110	1
Light + H_2_O_2_	180	27
Light + H_2_O_2_ + PANI	170	49
Light + H_2_O_2_ + CuFe_2_O_4_	150	55
Light + H_2_O_2_ + 5% PCF	90	87
Light + H_2_O_2_ + 10% PCF	75	95
Light + H_2_O_2_ + 15% PCF	75	96

#### 2.2.1 Effect of Irradiation Time

**Figure 9:**
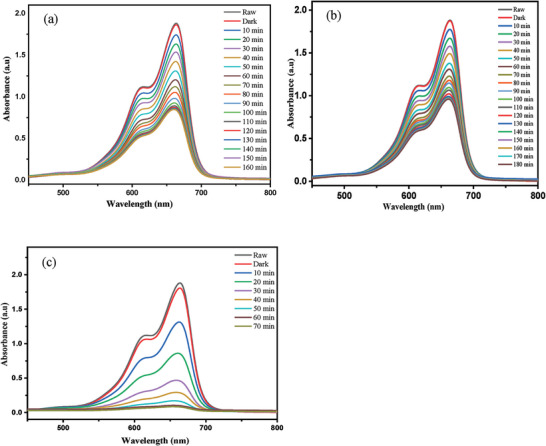
UV–Vis spectral changes of MB dye photodegradation by (a) PANI (b) CuFe_2_O_4_, and (c) 10% PANI‐CuFe_2_O_4_ nanocomposite.

The effect of irradiation time on photodegradation of MB dye was investigated under optimized condition, including PANI, CuFe_2_O_4_, as well as 5, 10, and 15% PCF nanocomposite under similar conditions (**Figure** **9**). The progress of the photocatalytic process was monitored by Ultraviolet‐visible (UV‐Vis) spectroscopy. The degradation reaction was well studied and revealed that composite material attained equilibrium within 70 min with a maximum percentage of degradation to be 95%, compared to individual materials such as PANI and CuFe_2_O_4_ that only contributed 33% and 39%, respectively.

#### 2.2.2 Effect of Initial Dye Concentration

**Figure 10:**
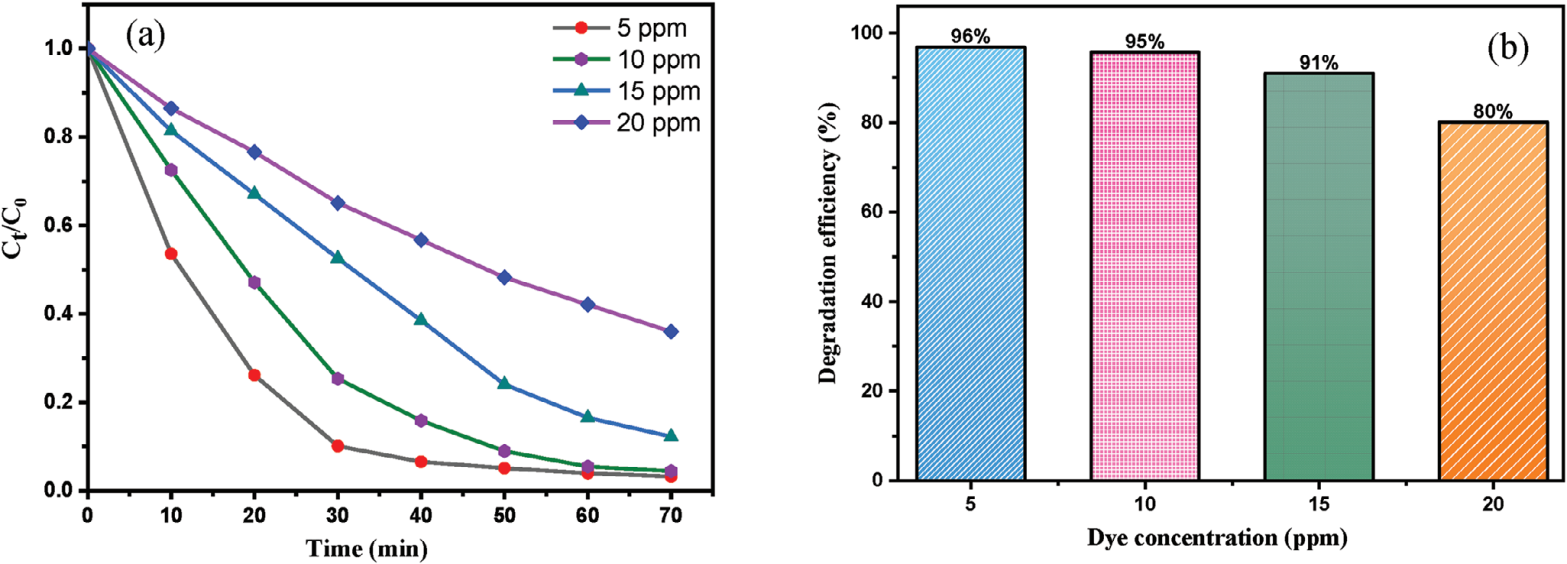
(a) Effect of MB dye concentration on photodegradation, and (b) variation of degradation efficiency with increasing concentration of the dye.

The effect of the initial dye concentration on the photocatalysis reaction was carried out by varying the concentration from 5  to 20 ppm under optimum condition (**Figure** **10**). The maximum performance of the photocatalyst attained up to 96% within 70 min at low concentration (5 ppm). In addition, the percentage surprisingly diminished to 80% at 20 ppm dye solution in 115 min. The efficacy of catalyst with increasing concentration significantly lowered owing to intermediates formation that considerably reduced the number of photons and hydroxyl radicals on the photocatalyst surface.^[^ ^35^, ^36^ ^]^

#### 2.2.3 Effect of Photocatalytic Dose

**Figure 11:**
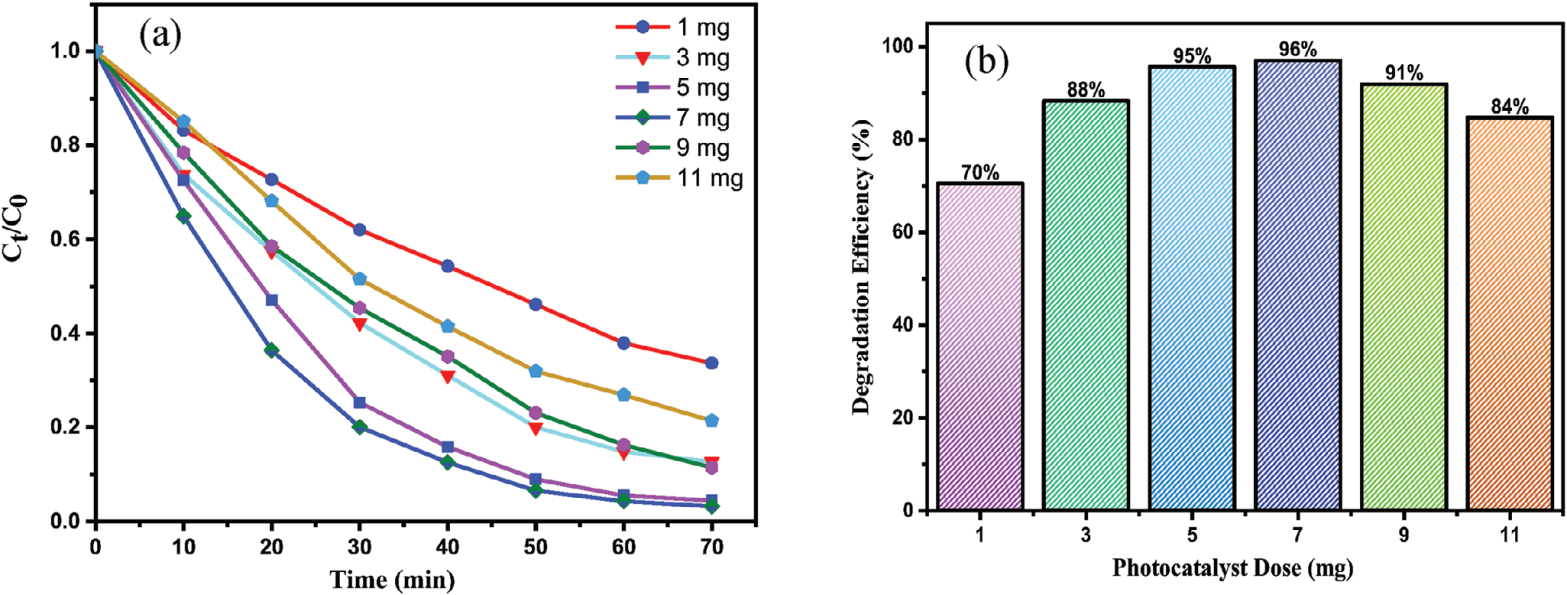
(a) Effect of 10% PANI‐CuFe_2_O_4_ composite dosage on photodegradation rates of MB, and (b) Variation of degradation efficiency with increasing dosages of nanocomposite.

The efficiency of photocatalyst to degrade MB was carefully investigated considering the different amounts of catalyst (1 to 7 mg) at a fixed concentration. **Figure** **11**b expressed an escalated percentage of dye degradation from 70% to 96% by increasing the amount from 1 to 7 mg owing to the generation of more •OH radicals. However, the percentage lessened to 84% in presence of 11 mg of catalyst due to unavailability of active sites on the catalyst surface.^[^ ^37^, ^38^ ^]^ Besides, excessive amounts of photocatalyst enhance the opacity of the suspension as well as impede the passage of visible light into the suspension that decreasing the activity of the photodegradation rate.^[^ ^39^ ^]^

### 2.3 Kinetic Study

**Figure 12:**
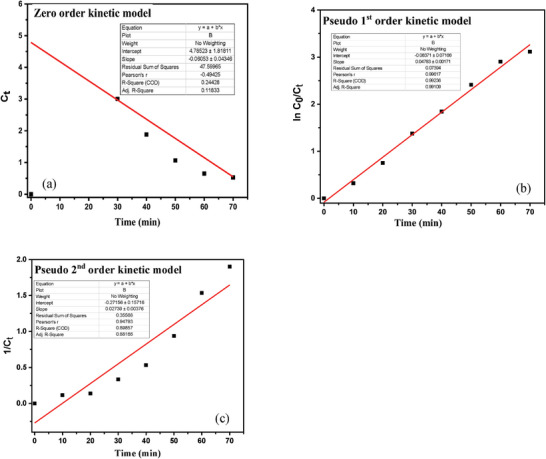
Fitted plotes of different Kinetic model of photocatalytic degradation reaction of MB dye.

The kinetic study of photocatalytic degradation of MB is an important measurement that suggests the efficiency of photocatalyst. In the present study, three kinetic models, including zero order, pseudo‐first‐order kinetic and pseudo‐second‐order kinetic were utilized to evaluate the performance of the photocatalyst shown in **Table** **3**. The given graphical (**Figure** **12**) representation of ln (C_0_/C_t_) with time (t) elicits the straight line with the highest R^2^ value (0.99081), suggesting of good fit for Pseudo‐first‐order, and the rate constant, k, was found to be 0.04784 min^−1^.

**Table 3:** Kinetic model for photodegradation reaction of MB.

Kinetic model	Equation	R^2^ value	Rate constant (k)	
Zero order	C_0_ = C_t_ − k_0_t	0.24	0.06053 mol L^−1^ min^−1^	[37]
Pseudo‐first‐ order	$\ln\;\frac{C_{0}}{C_{t}}=-\mathrm{kt}$	0.99	0.04783 min^−1^	
Pseudo‐second‐order	$\frac{1}{C_{t}}=\frac{1}{C_{0}}+\mathrm{kt}$	0.88	0.02739 M^−1^ min^−1^	

### 2.4 Proposed Mechanism of Photocatalytic Degradation of MB

**Figure 13:**
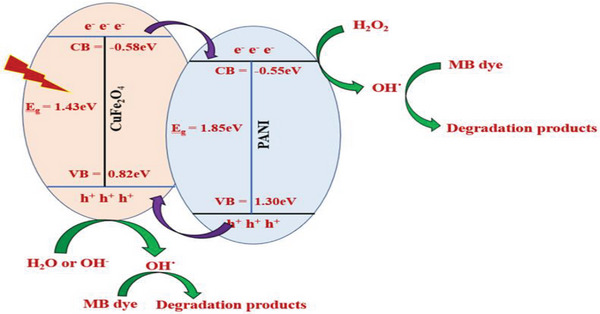
Proposed mechanism of photocatalytic MB dye degradation by PANI‐CuFe_2_O_4_ nanocomposite.

The possible mechanism of photocatalytic degradation of MB has been highlighted in **Figure** **13**. In this present study, the degradation efficiency of the PCF nanocomposite might be increased due to the significant decrease in charge transfer resistance in the composite compared to the naked CuFe_2_O_4_. Since the photogenerated electrons from the conduction band (CB) of CuFe_2_O_4_ nanoparticles can transfer easily to the CB of PANI, which has superior electrical conductivity, effectively preventing a direct recombination of electrons and holes leading to enhancement in the MB dye degradation. Furthermore, the enhanced activity of the composite is attributed to the synergistic effects of the heterostructures, efficient visible light utilization, and improved stability. In photocatalytic dye degradation experiments, visible light irradiation generates electrons in the CB and holes in the valence band (VB). In this study, CuFe_2_O_4_ absorbs light more efficiently than PANI under visible light irradiation due to its lower bandgap, generating more charges in the VB and CB. Also, the VB of CuFe_2_O_4_ has a lower positive potential, and the CB has a higher negative potential compared to PANI.^[^ ^40^ ^]^ Therefore, electrons in the CB of CuFe_2_O_4_ transfer to the CB of PANI, while holes from PANI migrate to the VB of CuFe_2_O_4_. The photogenerated electron‐hole pairs move to active sites at the semiconductor/liquid interface, reacting with adsorbed species. These photogenerated holes and electrons act as strong oxidizing and reducing agents, triggering subsequent oxidative and reductive reactions. The electrons injected into the CB of PANI reduce adsorbed oxygen to form reactive superoxide anions (O_2_^−^˙), which further generates hydroxyl radical (OH˙). Meanwhile, holes in the VB of PANI and CuFe_2_O_4_ oxidize organic species or form active OH˙ by decomposing water or reacting with hydroxyl ions (OH^−^). OH˙, a powerful oxidant and O_2_^−^˙ break down MB dye into CO_2_, H_2_O, and other stable byproducts.^[^ ^10^, ^41^ ^]^

Visible light irradiation:

(2) $$\mathrm{PCF}+\mathrm{hv}\;\;\left(\mathrm{light}\mathrm{irradiation}\right)\to \left\{e^{-}\left(\mathrm{CB}\right)+h^{+}\left(\mathrm{VB}\right)\right\}$$

Photo‐reduction reaction:

(3) $$\left\{e^{-}\left(\mathrm{CB}\right)+h^{+}\left(\mathrm{VB}\right)\right\}\to \mathrm{PANI}\{e^{-}\left(\mathrm{CB}\right)\}+\mathrm{CuF}e_{2}O_{4}\left\{h^{+}\left(\mathrm{VB}\right)\right\}$$

(4) $$\mathrm{PANI}\{e^{-}\left(\mathrm{CB}\right)\}+O_{2}\to {O_{2}}^{-\cdot }\to OH^{\cdot }$$

(5) $$\mathrm{MB}\mathrm{Dye}+OH^{\cdot }\to CO_{2}+H_{2}O+\mathrm{Other}\mathrm{degradation}\mathrm{products}$$

Photo‐oxidation reaction:

(6) $$\mathrm{CuF}e_{2}O_{4}\left\{h^{+}\left(\mathrm{VB}\right)\right\}+H_{2}O\to OH^{\cdot }+H^{+}$$

(7) $$\mathrm{CuF}e_{2}O_{4}\left\{h^{+}\left(\mathrm{VB}\right)\right\}+OH^{-}\to OH^{\cdot }$$

(8) $$\begin{array}{ccc} & & \mathrm{MB}\mathrm{Dye}+OH^{\cdot }/\mathrm{PCF}\{h^{+}\left(\mathrm{VB}\right)\}CO_{2}+H_{2}O\\ & & \;+\;\mathrm{Other}\;\mathrm{degradation}\;\mathrm{products} \end{array}$$

Moreover, in this study, hydrogen peroxide (H_2_O_2_) created a Fenton‐like catalyst system, enhancing MB dye degradation by forming OH˙ and HO2˙. The ferrocenyl group reacts with H_2_O_2_, generating ferrocenium cation and OH˙, which are regenerated by electron transfer from H_2_O_2_, producing HO2˙. The self‐redox properties of copper and iron in CuFe_2_O_4_, induced by H_2_O_2_, further facilitate OH˙ generation. Both OH˙ and HO_2_˙ are key oxidative species responsible for MB degradation.^[^ ^42^, ^43^ ^]^

### 2.5 Recyclability

**Figure 14:**
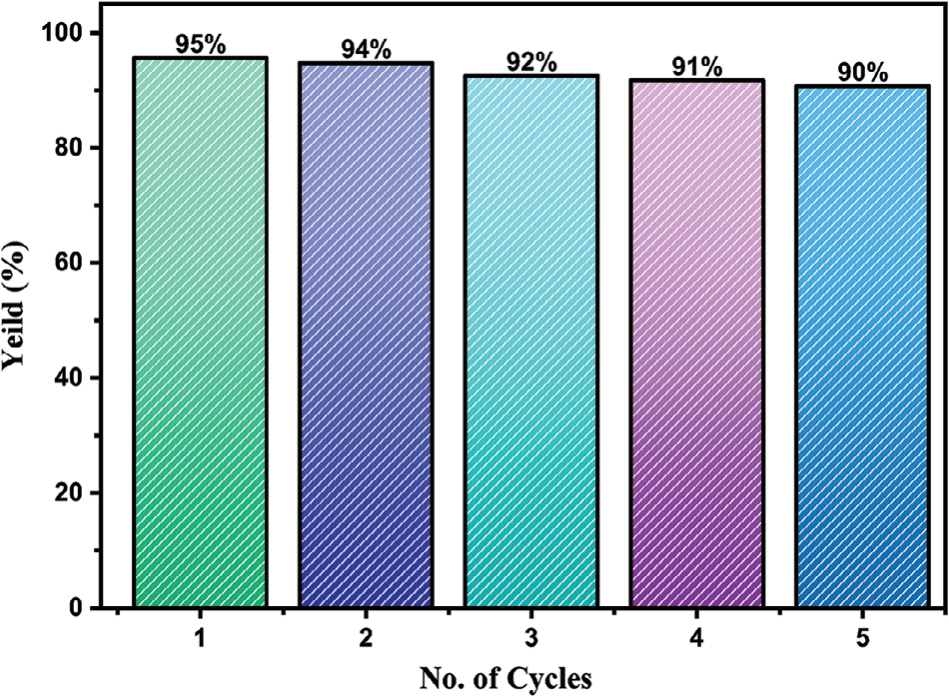
Reusability of PANI‐CuFe_2_O_4_ nanocomposite in MB dye photodegradation.

Recyclability is an important parameter for evaluating the performance of photocatalysts (**Figure** **14**). The recyclability experiment was conducted for five consecutive cycles, and the degradation performance varied from 95% to 90%, resulting the decrease of gradual performance owing to the clogging of active sites on the photocatalyst that impeded the **effective** combination of dye molecules on the catalyst surface.^[^ ^67^ ^]^ Nevertheless, it might be certainly inferred that the stability and reusability of synthesized nanocomposite in this study is comparable with reported works as shown in Table 4.

**Table 4:** Photodegradation of MB dye using polymer‐based composites.

Catalyst	Dye concentration (ppm)	Catalyst dose (mg)	Irradiation source	Degradation (%)	Irradiation time (min)	Refs.
PANI/NiFe_2_O_4_	30	400	Visible light	88	120	[11]
Chitosan ‐grafted‐PANI/Co_3_O_4_	10	15	UV lamp	88	180	[14]
PANI/Bi_2_SnTiO_7_	8	30	Visible light	100	220	[45]
PANI/g‐C_3_N_4_	10	100	Visible light	92	120	[17]
PANI/g‐C_3_N_4_	8	250	Visible light	90–98	40	[46]
PANI/Ceria	10	30	Visible light	56	150	[47]
PANI/NiO	10	100	Visible light	76	300	[48]
PANI/ZnO	3.2	1	UV light and sunlight	79 (UV), 97 (sunlight)	300	[49]
PANI/ZnO	50	–	Visible light	58.9	120	[50]
PANI/CdO	4.8	20	UV light, sunlight	92 (UV), 98 (sunlight)	240	[26]
PANI/TiO_2_	10	50	Visible light	81	120	[51]
PANI/TiO_2_	63.8	100	Visible light	77	120	[52]
PANI/SrTiO_3_	10	30	Visible light	97	90	[53]
PANI/Bi_2_O_3_	5	100	Visible light	96	150	[54]
PANI/BiVO_4_/GO	3.2	100	Visible light	73	180	[55]
PANI/BiVO_4_/GO	–	100	Visible light	73	120	[56]
PANI/WO_3_	20	16.4	Visible light	88	180	[57]
WS_2_/PANI	10	20	UV light	99	90	[58]
PPy/g‐C_3_N_4_	10	50	Visible light	99	120	[59]
PEG/TiO_2_	20	3000	UV light	100	240	[60]
PCTFE/TiO_2_	10	–	UV light	100	270	[61]
PPy/ZnO	50	50	UV light	98	20	[62]
PVA/g‐PAM/ZnO/SiO_2_	5	100	UV light	86	960	[63]
PUF/rGO/ZnO	–	–	Visible light	100	180	[64]
PMMA/ZnO	4.8	100	UV light	60	240	[65]
Cellulose acetate/ZnO	50	10	UV light, sunlight	30 (UV), 75 (sunlight)	120	[66]
PANI/CuFe_2_O_4_	10	5	Visible light	96	75	This study

### 2.6 Phytotoxicity Assay

**Figure 15:**
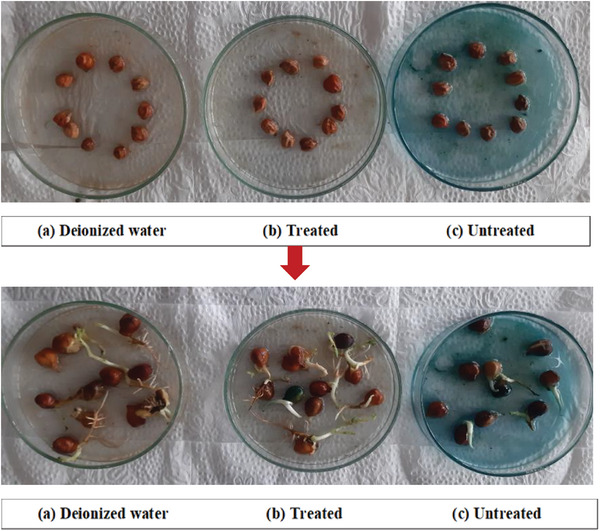
Phytotoxicity test of control water, treated, and untreated MB dye solution using chickpea seeds.

**Figure 16:**
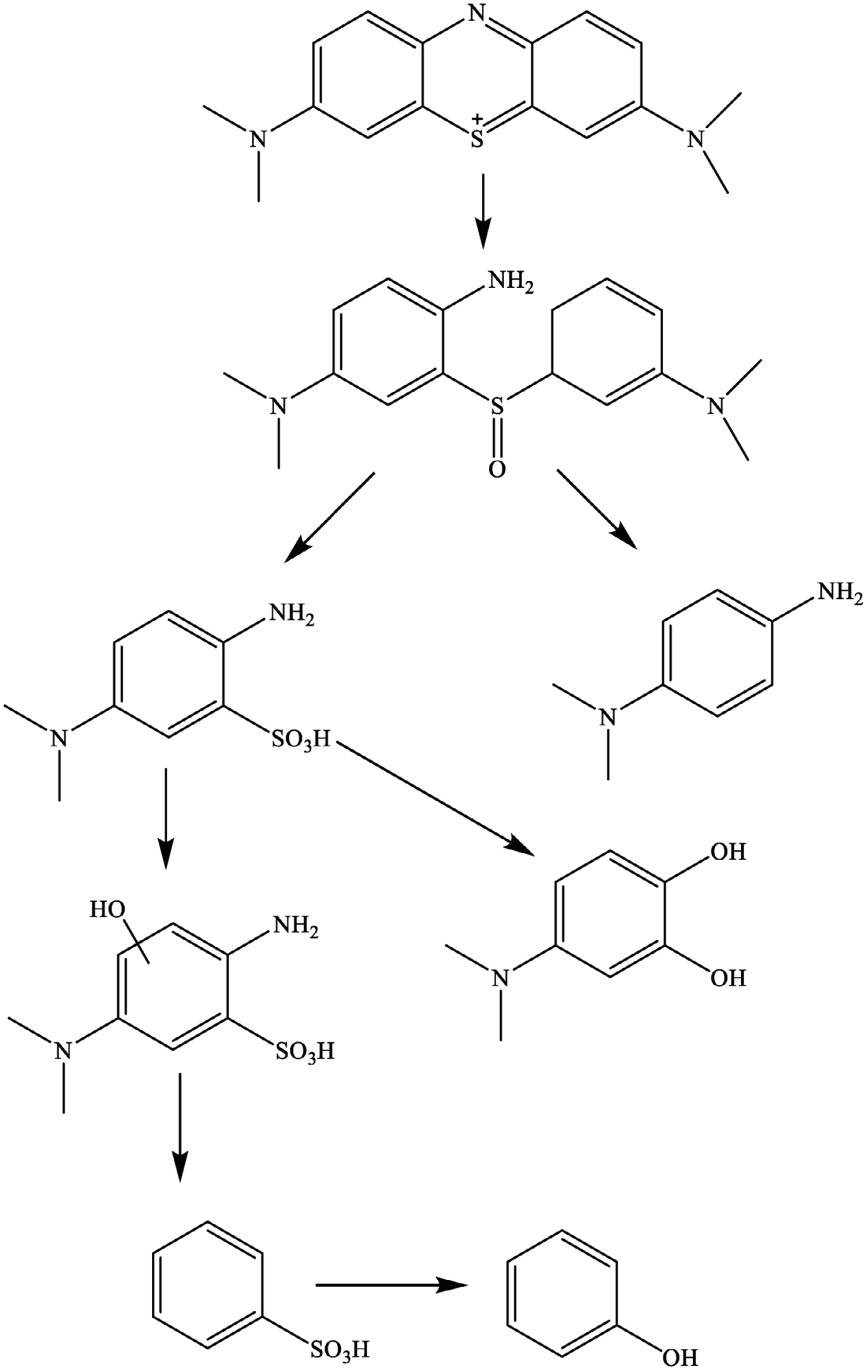
Proposed structural change of MB dye during photodegradation.^[^ ^44^ ^]^

Photocatalytic degradation of organic compounds such as dyes converts into hazardous intermediates or by products that exert adverse impacts on biota. To ascertain the toxicity of MB dye and associated compounds, bioassay approach was conducted considering untreated dye solution, treated dye solution, and deionized water (**Figure** **15**).^[^ ^68^ ^]^ However, the notable percentage of germination was found to be 90% by deionized water, but the germination rate was significantly reduced to 40% in the presence of untreated dye water, along with shorter root and shoot lengths (**Table** **5**). In addition, the treated dye water ameliorated the germination percentage up to 80%, suggesting the nanocomposite degraded MB dye into less toxic components supported by the previous study as depicted in **Figure** **16**.

**Table 5:** Phytotoxicity of control water, treated and untreated MB dye solution using chickpea seeds.

Parameters	Germination [%]	Root length [cm]	Shoot length [cm]	Germination index [%]
Control water	90	2.04 ± 1.11	2.21 ± 1.46	100
Treated dye solution	80	1.88 ± 0.88	2.06 ± 1.12	81.92
Untreated dye solution	40	0.18 ± 0.02	1.33 ± 0.86	3.92

## 3 Conclusion

Heterostructure semiconductor type photocatalysis has emerged as a highly promising avenue for the degradation of organic dyes. In this context, light responsive photocatalysts, PANI‐CuFe_2_O_4_, have successfully been synthesized by sol–gel method. An array of analytical techniques was employed for the characterization of composite material. Of them, FTIR analysis unveiled the presence of M─O, while XRD analysis offered invaluable insights about crystal size of 39.94 nm with crystalline in nature. EDX study has also supported the confirmation of nanocomposite formation. The photocatalytic performance of PANI‐CuFe_2_O_4_ has been extensively studied under UV light irradiation, in which maximum efficacy attained 96% within a short time span. The significant outcome of the research is the durability and better performance of the catalyst even after several cycles. The kinetic investigation explores the photocatalytic process and proceeds with the supporting of pseudo‐second order, exhibiting the highest correlation coefficient value (R^2^  =  0.99).

## 4 Experimental Section

### Chemicals

All chemicals such as copper (II) nitrate trihydrate, ferric nitrate nonahydrate, sodium hydroxide, aniline, hydrochloric acid, ammonium peroxydisulfate, ethanol, and deionized water were purchased from Merck, India, and all the chemicals were in analytical grade and used without further purification. Double deionized water was used throughout the experiment.

### Synthesis of CuFe_2_O_4_

The nanocatalyst, CuFe_2_O_4_ was prepared using co‐precipitation method. Stoichiometric proportion of aqueous solution of copper (II) nitrate trihydrate and ferric nitrate nonahydrate were mixed and kept under ultrasonication for 1 h at room temperature. Following this, NaOH (4M) was added drop wish to reach pH of solution until 12, and stirring continued for 2 h followed by keeping in steady state for overnight. Eventually, brown color precipitate was obtained, and it was successively washed for three times with ethanol and deionized water followed by centrifugation. The resulting CuFe_2_O_4_ was desiccated at 100 °C for 8 h and ground homogeneously. The powder was calcinated at 850 °C for 2 h for further investigation.

### Synthesis of PANI

PANI was synthesized using chemical oxidative polymerization. Hydrochloric acid (150 mL of 1 m) was taken, and monomer aniline (4.5 mL) was added slowly with continuous stirring keeping in an ice bath. Ammonium peroxydisulfate solution (2g in 50 mL of deionized water) was added to the mixture dropwise with continuous stirring for 2 h. The mixture was allowed for constant stirring for 24 h to complete the polymerization, followed by keeping for 12 h in a steady state. Finally, the green precipitate was isolated from the reaction system that was washed several times with deionized water. After that, the desired substance was dried at 120 °C for 24 h, and was ground for further experiment.

### Synthesis of PANI‐CuFe_2_O_4_

The catalyst, PCF, was synthesized using wet impregnation method. For the preparation of 10% PCF nanocomposite, a fixed amount of (600 mg) CuFe_2_O_4_ was immersed in ethanol (50 mL), and it was sonicated for 4 h to dispersed homogeneously in ethanol. A specific amount of PANI (60 mg) was added to CuFe_2_O_4_ (600 mg), and the mixture was stirred again at constant stirring for 24 h. The PANI coated CuFe_2_O_4_ was dried at 100 °C for 8 h, and was stored in airtight condition after grinding. The same procedure was repeated for the preparation of 5% and 15% nanocomposites by varying the amount of PANI.

### Characterizations of Photocatalysts

The synthesized photocatalysts were characterized in several spectroscopic and analytical techniques, including UV–vis spectrophotometer (UV‐1900i, SHIMADZU, Japan), Fourier Transform Infrared spectrophotometer (FT‐IR) (IRSpirit, SHIMADZU, Japan) using a KBr pellet with a scan rate of ≈4 cm s^−1^ at 25 °C, scanning electron microscopy (SEM), energy dispersive X‐ray spectroscopy (EDX) analysis (SU‐8000, Hitachi, Japan) at accelerating voltages of 10 and 15 kV, and X‐ray diffraction (XRD) (Rigaku Smart Lab spectrometer, Japan) with Cu‐Kα radiation. Thermo gravimetric analysis (TGA) was carried from 25 to 800 °C (Heating rate, 10 °C min^−1^) under inert atmosphere (N_2_ gas, Flow rate: 2 mL min^−1^) using a thermo gravimetric analyzer (TGA‐50 SHIMADZU, Japan).

### Evaluation of Catalytic Performance

Batch experiments were conducted at room temperature to investigate the catalytic performance of photocatalyst. In order to achieve adsorption‐desorption equilibrium, the reaction was carried out in dark for the first 30 min, and after that, the photocatalytic dye degradation experiments were carried out under visible light (100w Philips bulb; 220–240 V) irradiation in the presence of H_2_O_2_. The change of absorbance was measured using UV–vis spectrophotometer at 664 nm in regular time intervals. The photocatalytic performance of PANI, CuFe_2_O_4_, and PCF was optimized considering the parameters, including contact time, concentration of dye, and dosage of the composite. The kinetic of the catalysis was examined to find out the optimum condition of MB dye photodegradation. The percentage of dye degradation was calculated through the following equation.^[^ ^69^ ^]^

(9) $$Photodegradation\;\left(\%\right)=\frac{C_{0}-C_{t}}{\;C_{0}}\times 100\%$$

### Phytotoxicity Study

Phytotoxicity of intermediates or degraded by‐products originating from MB dye degradation was carried out considering the model seed of *Cicer arietinum*, known as Chickpea. The seeds were kept in untreated, treated, and controlled water containing petri dishes for germination at room temperature over a period of seven days. Eventually, germination rates, root lengths, and shoot lengths of Chickpea seeds were assessed after 7 days. The percentage of germination and germination index was computed in the following equations.^[^ ^45^, ^46^ ^]^

(10) $$\begin{array}{ccc} \mathrm{Percentage}\;\mathrm{of}\;\mathrm{germination}\;\left(\%\right) & = & \frac{No\;of\;seeds\;germinated\;in\;sample}{No\;of\;seeds\;sowed}\\ & & \times \;100\% \end{array}$$

(11) $$\begin{array}{ccc} & & \mathrm{Percentage}\;\mathrm{of}\;\mathrm{germination}\;\mathrm{index}\;\left(\%\right)\\ & & \;=\frac{Seed\;germination\;\left(\%\right)\;\times \;Root\;length\;of\;treatment}{Seed\;germination\;\left(\%\right)\times Root\;length\;of\;control}\times \;100\% \end{array}$$

## Conflict of Interest

The authors declare no conflict of interest.

## Author Contributions

This work was conducted by all authors.

## Data Availability

The data that support the findings of this study are available from the corresponding author upon reasonable request.

## References

[1] K. Badvi , V. Javanbakht , J. Cleaner. Prod. 2021, 280, 124518.

[2] D. Gogoi , P. Makkar , N. N. Ghosh , ACS Omega 2021, 6, 4831. 33644591 10.1021/acsomega.0c05809 PMC7905952

[3] S. Yesmin , M. Mahiuddin , A. B. M. Nazmul Islam , K. M. R. Karim , P. Saha , M. A. R. Khan , H. M. Ahsan , ACS Omega 2024, 9, 10727. 38463303 10.1021/acsomega.3c09557 PMC10918656

[4] V. Katheresan , J. Kansedo , S. Y. Lau , J. Environ. Chem. Eng. 2018, 6, 4676.

[5] R. Kodasma , B. Palas , G. Ersöz , S. Atalay , Ceram. Int. 2020, 46, 6284.

[6] N. Jaafarzadeh , F. Ghanbari , M. Ahmadi , Chem. Eng. J. 2017, 320, 436. 10.1016/j.chemosphere.2016.11.038 27898330

[7] Y. Zhao , C. Lin , H. Bi , Y. Liu , Q. Yan , Appl. Surf. Sci. 2017, 392, 701.

[8] Q. Mo , S. Zeng , J. Yang , C. Wu , Y. Zhang , J. Ceram. Soc. Jpn. 2020, 128, 135.

[9] L. Wei , Y. Zhang , S. Chen , L. Zhu , X. Liu , L. Kong , L. Wang , J. Environ. Sci. 2019, 76, 188. 10.1016/j.jes.2018.04.024 30528009

[10] P. Xiong , L. Wang , X. Sun , B. Xu. X. Wang , Ind. Eng. Chem. Res. 2013, 52, 10105.

[11] M. R. Patil , V. Shrivastava , Der. Chemica. Sinica. 2014, 5, 8.

[12] S. Sharma , A. Kaur , Indian. J. Sci. Technol. 2018, 11, 1.

[13] X. Tan , X. Tan , J. Wang , X. Pang , L. Liu , Q. Sun , Q. You , F. Tan , N. Li , ACS Appl. Mater. Interfaces. 2016, 8, 34991. 27957854 10.1021/acsami.6b11262

[14] S. Shahabuddin , N. M. Sarih , F. H. Ismail , M. M. Shahid , N. M. Huang , RSC Adv. 2015, 5, 83857.

[15] P. Yadav , P. K. Surolia , D. Vaya , Mater. Today: Proc. 2021, 43, 2949.

[16] Y. You‐Yi , Z. Heng‐Qiang , Chin. J. Struct. Chem. 2016, 35, 472.

[17] L. Ge , C. Han , J. Liu , J. Mater. Chem. 2012, 22, 11843.

[18] S. Hassan , A. H. Kamel , A. A. Hassan , A. Amr , H. Abd El‐Naby , M. Al‐Omar , A. Sayed , Molecules 2020, 25, 2721. 32545457 10.3390/molecules25122721 PMC7356621

[19] V. J. Babu , S. Vempati , S. Ramakrishna , Mater. Sci. App. 2013, 4, 27057.

[20] A. John , S. K. Mahadeva , J. Kim , Smart Mater. Struct. 2010, 19, 045011.

[21] S. Sultana , M. Z. Khan , K. Umar , J. Alloys. Compd. 2012, 535, 44.

[22] P. Kharazi , R. Rahimi , M. Rabbani , Solid State Sci. 2019, 93, 95.

[23] Y. Shen , Y. Wu , H. Xu , J. Fu , X. Li , Q. Zhao , Y. Hou , Mater. Res. Bull. 2013, 48, 4216.

[24] J. Calvo‐de la Rosa , M. Segarra , ACS Omega 2019, 4, 18289. 31720529 10.1021/acsomega.9b02295 PMC6844087

[25] D. Gupta , V. Rishi , T. K. Gupta , Mater. Res. Innovations. 2020, 25, 393.

[26] H. Gülce , V. Eskizeybek , B. Haspulat , F. Sarı , A. Gülce , A. Avcı , Ind. Eng. Chem. Res. 2013, 52, 10924.

[27] R. S. Yadav , I. Kuřitka , J. Vilcakova , J. Havlica , J. Masilko , L. Kalina , J. Tkacz , M. Hajdúchová , V. Enev , J. Mater. Sci.: Mater. Electron. 2017, 28, 6245.

[28] M. Salavati‐Niasari , T. Mahmoudi , M. Sabet , S. M. Hosseinpour‐Mashkani , F. Soofivand , F. Tavakoli , J. Cluster. Sci. 2012, 23, 1003.

[29] M. Mosabberul Haque , A. Rahman , M. Shafiul Islam Shahin , M. Ahsan Habib , M. Abu Rayhan Khan , M. Mahiuddin , M. Uddin Monir , K. Md Rezaul Karim , Results. Chem. 2024, 7, 101509.

[30] S. Saha , N. Chaudhary , A. Kumar , M. Khanuja , SN Appl. Sci. 2020, 2, 1115.

[31] Y. Mosqueda , E. Pérez‐Cappe , J. Arana , E. Longo , A. Ries , M. Cilense , P. A. P. Nascente , P. Aranda , E. Ruiz‐Hitzky , J. Solid. State. Chem. 2006, 179, 308.

[32] Y. Yao , F. Lu , Y. Zhu , F. Wei , X. Liu , C. Lian , S. Wang , J. Hazard. Mater. 2015, 297, 224. 25974659 10.1016/j.jhazmat.2015.04.046

[33] J. A. Jiménez‐Miramontes , J. L. Domínguez‐Arvizu , F. A. Gaxiola‐Cebreros , B. C. Hernández‐Majalca , J. C. Pantoja‐Espinoza , J. M. Salinas‐Gutiérrez , V. H. Collins‐Martínez , A. López‐Ortiz , Rev. Adv. Mater. Sci. 2022, 61, 654.

[34] B. Thomas , L. Alexander , Appl. Nanosci. 2018, 8, 125.

[35] I. K. Konstantinou , T. A. Albanis , Appl. Catal., B 2004, 49, 1.

[36] S. S. Sambaza , A. Maity , K. Pillay , ACS Omega 2020, 5, 29642. 33251400 10.1021/acsomega.0c00628 PMC7689664

[37] S. Malato , P. Fernández‐Ibáñez , M. I. Maldonado , J. Blanco , W. Gernjak , Catal. Today 2009, 147, 1.

[38] P. Jantawasu , T. Sreethawong , S. Chavadej , Chem. Eng. J. 2009, 155, 223.

[39] H. Zhu , R. Jiang , Y. Fu , Y. Guan , J. Yao , L. Xiao , G. Zeng , Desalination 2012, 286, 41.

[40] K. M. R. Karim , M. Tarek , S. M. Sarkar , R. Mouras , H. R. Ong , H. Abdullah , C. K. Cheng , M. M. R. Khan , Int. J. Hydrogen Energy 2021, 46, 24709.

[41] A. Ajmal , I. Majeed , R. N. Malik , H. Idriss , M. A. Nadeem , RSC Adv. 2014, 4, 37003.

[42] E. Neyens , J. Baeyens , J. Hazard. Mater. 2003, 98, 33. 12628776 10.1016/s0304-3894(02)00282-0

[43] A. Nawaz , A. Khan , N. Ali , N. Ali , M. Bilal , Environ. Technol. Innovation. 2020, 20, 101079.

[44] A. Houas , Appl. Catal., B 2001, 31, 145.

[45] Y. Yang , J. Luan , Molecules 2012, 17, 2752. 22395405 10.3390/molecules17032752 PMC6268818

[46] H.‐H. Wu , C.‐W. Chang , D. Lu , K. Maeda , C. Hu , ACS Appl. Mater. Interfaces. 2019, 11, 35702. 31532604 10.1021/acsami.9b10555

[47] J. Vidya , P. Balamurugan , Desalination. Water Treatment 2019, 156, 349.

[48] J. Vidya , P. Balamurugan , Mater. Res. Express. 2019, 6, 0950c8.

[49] V. Eskizeybek , F. Sari , H. Gülce , A. Gülce , A. Avci , Appl. Catal. B: Environ. 2012, 119, 197.

[50] R. Qin , L. Hao , Y. Liu , Y. Zhang , Chem. Select 2018, 3, 6286.

[51] F. Wang , S. Min , Y. Han , L. Feng , Superlattices Microstruct. 2010, 48, 170.

[52] E. Subramanian , S. Subbulakshmi , C. Murugan , Mater. Res. Bull. 2014, 51, 128.

[53] S. Shahabuddin , N. Muhamad Sarih , S. Mohamad , J. Joon Ching , Polymers 2016, 8, 27. 30979143 10.3390/polym8020027 PMC6432585

[54] B. Li , Y. Li , Y. Kang , Mater. Lett. 2021, 286, 129226.

[55] M. R. U. D. Biswas , K. Y. Cho , J. D. Na , W.‐C. Oh , Mater. Sci. Eng.: B 2019, 251, 114469.

[56] M. R. U. D. Biswas , B. S. Ho , W.‐C. Oh , Polym. Bull. 2019, 77, 4381.

[57] X. Jia , D. Jiang , P.‐I. Gouma , Mater. Lett. 2022, 314, 131869.

[58] V. Sodha , S. Shahabuddin , R. Gaur , I. Ahmad , R. Bandyopadhyay , N. Sridewi , Nanomaterials 2022, 12, 3199. 36144986 10.3390/nano12183199 PMC9504493

[59] H. Han , M. Fu , Y. Li , W. Guan , P. Lu , X. Hu , Chin. J. Catal. 2018, 39, 831.

[60] Y. Liu , X. Li , M. Xing , J. Jin , J. Phys. Chem. Solids 2021, 158, 110231.

[61] X. Chen , X. Li , Y. Li , L. Zhao , Y. Sun , I. E. Rushimisha , T. Han , L. Weng , X. Lin , Y. Li , Environ. Technol. Innovation. 2021, 24, 101901.

[62] A. R. Singh , P. S. Dhumal , M. A. Bhakare , K. D. Lokhande , M. P. Bondarde , S. Some , Sep. Purif. Technol. 2022, 286, 120380.

[63] P. Kongseng , P. Amornpitoksuk , S. Chantarak , React. Funct. Polym. 2022, 172, 105207.

[64] M. Morsy , A. I. Abdel‐Salam , I. Gomaa , H. Moustafa , H. Kalil , A. Helal , Molecules 2022, 28, 108. 36615302 10.3390/molecules28010108 PMC9822506

[65] A. Di Mauro , M. Cantarella , G. Nicotra , G. Pellegrino , A. Gulino , M. V. Brundo , V. Privitera , G. Impellizzeri , Sci. Rep. 2017, 7, 40895. 28098229 10.1038/srep40895 PMC5241647

[66] M. A. Abu‐Dalo , S. A. Al‐Rosan , B. A. Albiss , Polymers 2021, 13, 3451. 34641266 10.3390/polym13193451 PMC8512553

[67] R. S. Brishti , M. Ahsan Habib , M. H. Ara , K. M. Rezaul Karim , M. Khairul Islam , J. Naime , M. M. Hasan Rumon , M. A. Rayhan Khan , Results. Chem. 2024, 7, 101441.

[68] S. Ramanathan , N. Radhika , D. Padmanabhan , A. Durairaj , S. Paul Selvin , S. Lydia , S. Kavitha , S. Vasanthkumar , ACS Omega 2019, 5, 158. 31956762 10.1021/acsomega.9b02281 PMC6963968

[69] A. G. Naikwade , M. B. Jagadale , D. P. Kale , A. D. Gophane , K. M. Garadkar , G. S. Rashinkar , ACS Omega 2020, 5, 131. 31956760 10.1021/acsomega.9b02040 PMC6963935

